# Microstructural Neuroimaging of Frailty in Cognitively Normal Older Adults

**DOI:** 10.3389/fmed.2020.546344

**Published:** 2020-10-23

**Authors:** Qu Tian, Owen A. Williams, Bennett A. Landman, Susan M. Resnick, Luigi Ferrucci

**Affiliations:** ^1^Longitudinal Studies Section, National Institute on Aging, Baltimore, MD, United States; ^2^Laboratory of Behavioral Neuroscience, National Institute on Aging, Baltimore, MD, United States; ^3^Department of Experimental Psychology, University of Oxford, Oxford, United Kingdom; ^4^School of Engineering, Vanderbilt University, Nashville, TN, United States

**Keywords:** microstructure, neuroimaging, diffusion tensor imaging, frailty, cognitively normal

## Abstract

Physical frailty is an age-related clinical syndrome that is associated with multiple adverse health outcomes, including cognitive impairment and dementia. Recent studies have shown that frailty is associated with specific volumetric neuroimaging characteristics. Whether brain microstructural characteristics, particularly gray matter, associated with frailty exist and what their spatial distribution is have not been explored. We identified 670 participants of the Baltimore Longitudinal Study of Aging who were aged 60 and older and cognitively normal and who had concurrent data on frailty and regional microstructural neuroimaging markers by diffusion tensor imaging (DTI), including mean diffusivity (MD) of gray matter and fractional anisotropy (FA) of white matter. We identified neuroimaging markers that were associated with frailty status (non-frail, pre-frail, frail) and further examined differences between three groups using multivariate linear regression (non-frail = reference). Models were adjusted for age, sex, race, years of education, body mass index, scanner type, and Apolipoprotein E e4 carrier status. Compared to the non-frail participants, those who were frail had higher MD in the medial frontal cortex, several subcortical regions (putamen, caudate, thalamus), anterior cingulate cortex, and a trend of lower FA in the body of the corpus callosum. Those who were pre-frail also had higher MD in the putamen and a trend of lower FA in the body of the corpus callosum. Our study demonstrates for the first time that the microstructure of both gray and white matter differs by frailty status in cognitively normal older adults. Brain areas were not widespread but mostly localized in frontal and subcortical motor areas and the body of the corpus callosum. Whether changes in brain microstructure precede future frailty development warrants further investigation.

## Introduction

Physical frailty is an age-related clinical syndrome that has been both cross-sectionally and prospectively associated with multiple adverse health outcomes, such as disability, hospitalization, and mortality, but in the initial stage, it is thought to be potentially reversible (i.e., pre-frail stage) (1–4). Most research on frailty has been focused on the role of reduced physiological reserve in cardiovascular, skeletomuscular, and immune systems. However, there is also evidence that changes in the central nervous system may also contribute or evolve in parallel to frailty.

Neuroimaging studies have shown that cerebrovascular damage, gray matter atrophy, as well as brain β-amyloid, may contribute to the pathophysiology of frailty. The severity of frailty status has been associated with a greater number of cerebral microbleeds (5), greater gray matter atrophy (6–8), higher white matter hyperintensity volume and infarcts (7–10), and higher brain β-amyloid burden (11, 12). A few longitudinal studies have shown that higher white matter hyperintensity volume or infarcts (13, 14), higher brain β-amyloid burden (11), and neuronal loss (14) are associated with the progression of frailty. A few studies examining regional brain atrophy with frailty suggest that brain areas important for motor function and execution may play a key role in frailty, such as selected areas in frontal, temporal, and parietal lobes, anterior cingulate cortex, subcortical areas, and cerebellum (6, 15).

Data on brain microstructure concerning frailty are sparse. Diffusion tensor imaging (DTI)-based brain microstructural measures may be sensitive to subtle age-related and vascular disease-related changes (16, 17) and can be used to monitor vascular disease progression (17). To date, only two DTI studies examined white matter microstructural characteristics with frailty (18, 19). These studies had relatively small samples and did not examine the microstructure of gray matter. Examining both gray and white matter microstructure across multiple regions of interest would allow us to determine the spatial distribution with frailty status, which may provide new insights into mechanisms and potential preventative strategies.

Examining sensitive neuroimaging markers, such as DTI-based microstructural integrity, and identifying specific brain areas connected with frailty or frailty risk in cognitively normal individuals may shed light on the spectrum of brain pathology underlying frailty. It may also identify new intermediate targets that can be tracked to verify the effectiveness of preventive interventions.

This exploratory study aimed to identify microstructural neuroimaging correlates of frailty, including both gray matter and white matter regions of interest (ROIs) in cognitively normal older adults. Our study poses an epidemiological question that is directly relevant for patient care: does DTI imaging provide important information to understand the contribution of the central nervous system to older patients' frailty status? We hypothesized that microstructural neuroimaging markers would be associated with frailty. We also hypothesized that microstructural correlates of frailty would be localized in brain areas important for motor function and execution, such as frontal, parietal and temporal lobes, anterior cingulate cortex, and subcortical areas.

## Methods

### Study Population

We identified 670 cognitively normal participants aged 60 and older from the Baltimore Longitudinal Study of Aging (BLSA) and used the first concurrent data on frailty status and microstructural neuroimaging markers of interest by DTI. BLSA is a prospective cohort study with continuous enrollment that began in 1958 (20). Data from this study were collected between June 2008 and December 2017. Exclusion criteria included diagnosis of mild cognitive impairment (MCI), Alzheimer's disease, and dementia. Diagnoses of cognitive impairment and dementia follow standard BLSA procedures, described previously (21). MCI was determined using the Peterson criteria (22). BLSA diagnose of dementia and Alzheimer's disease have continued to follow the Diagnostic and Statistical Manuel, third edition, revised (DSM-III-R) and the National Institute of Neurological and Communication Disorders and Stroke-Alzheimer's Disease and Related Disorders Association (NINCDS-ADRDA) criteria, respectively.

The BLSA protocol was approved by the Institutional Review Board of the National Institute of Environmental Health Sciences. Participants provided written informed consent at each BLSA visit.

### Frailty

We use the Fried criteria (23) and a previously identified population-independent cutoff (24) to determine frailty status, including non-frail (scored as 0), pre-frail (scored 1-2), and frail (scored 3-5). In brief, the frail status was defined when three or more of the following criteria were present: unintentional weight loss (≥ 10 lbs in the past year), self-reported exhaustion, slow gait speed, weakness (low grip strength), and low physical activity based on self-reported questionnaires (25). The previously validated population-independent cut-off approach has a good correlation and high agreement with the lowest-quintile approach (*r* = 0.84, weighted kappa = 0.75) (24). Because the BLSA sample tends to be healthier than the general population, we used 1.0 m/s to define slow gait speed (26). Components and cutoffs were presented in Supplementary Table 1.

### Imaging Acquisition

Imaging data were acquired on 3 T Philips Achieva scanner (scanners 1 and 2 at the Kennedy Krieger Institute and scanner 3 at the National Institute on Aging) in Baltimore, Maryland. Imaging evaluations for each participant included a T1-weighted magnetization-prepared rapid gradient-recalled echo (MPRAGE) scan, an interleaved proton density and T2-weighted dual-echo scan, a fluid-attenuated inversion recovery (FLAIR) scan, and two DTI scans. The MPRAGE protocol was as follows: number of slices = 170, voxel size = 1 mm × 1 mm × 1.2 mm, reconstruction matrix = 256 × 256, flip angle = 8 degrees and TR/TE = 6.5 ms/3.1 ms.

DTI acquisition protocol was identical for scanners 1 and 2: number of gradients = 32, number of b0 images = 1, max b-factor = 700 s/mm^2^, TR/TE = 6801/75 ms, number of slices = 65, voxel size = 0.83 × 0.83 × 2.2 mm, reconstruction matrix = 256 × 256, acquisition matrix = 96 × 95, field of view = 212 × 212 mm, flip angle = 90°.

DTI acquisition protocol for scanner 3 was different from scanners 1 and 2: number of gradients = 32, number of b0 images = 1, max b-factor = 700 s/mm^2^, TR/TE = 7454/75 ms, number of slices = 70, voxel size = 0.81 × 0.81 × 2.2 mm, reconstruction matrix = 320 × 320, acquisition matrix = 116 × 115, field of view = 260 × 260 mm, flip angle = 90°. Each DTI acquisition included two b0 images, which were averaged in k-space. The two separate DTI acquisitions with NSA = 1 were obtained and then combined offline (as explained in Image processing below) for an effective NSA = 2 to improve signal-to-noise ratio (27).

### Diffusion Tensor Imaging Processing

DTI processing follows the standard practice for tensor fitting and quality assessment and is explained in detail in earlier publications (21, 27). Briefly, the individual diffusion-weighted volumes were affine co-registered to a minimally weighted (b0) target to compensate for eddy current effects and physiological motion. The gradient tables were corrected for the identified rotational component using finite strain (28). To combine the two DTI sessions with different and unknown intensity normalization constants, each diffusion-weighted image was normalized by its own reference image before tensor fitting. QC was performed to remove scans with either excessive motion or images that had globally high diffusion measure bias after reviewing the distributions of QC summary statistics generated by our pipeline (27).

### Regions of Interest

In this study, we focused on fractional anisotropy (FA) of white matter ROIs and mean diffusivity (MD) of gray matter ROIs.

To segment gray matter regions, we used multi-atlas registration with the BrainCOLOR protocol using 35 manually labeled atlases from NeuroMorphometrics (29). The labels of ROIs obtained from the T1 image for each visit were affine registered to the diffusion image and used to extract region-specific average MD measures.

To segment white matter, the Eve White Matter atlas (30) was combined with corresponding WM labels from the multi-atlas segmentation (29), and an FA mapped MRI. The WM labels were then intersected with WM segmentation and the resulting labels are iteratively grown to fill the remaining WM space from the multi-atlas labels. The WM labels of ROIs obtained from the T1 image for each visit were affine registered to the FA image and used to extract region-specific average FA measures.

### Statistical Analysis

Univariate correlations of participant characteristics with frailty status (non-frail, pre-frail, frail) were examined using Spearman correlation coefficients for continuous variables and chi-square tests for categorical variables as appropriate.

We identified DTI-based neuroimaging markers that were being univariately associated with frailty status (non-frail, pre-frail, frail) using simple linear regression. We further adjusted for covariates that were important for brain health and/or frailty using multivariable linear regression. Covariates included age, sex, race, years of education, body mass index, and APOE e4 status.

For neuroimaging markers associated with frailty status at *p* ≤ 0.05, we further examined differences between non-frail, pre-frail, frail groups using multivariable linear regression with “non-frail” being the reference group, adjusting for age, sex, race, education, body mass index, APOE e4 status, and also the scanner type. Dummy coded vectors were created to define “pre-frail” and “frail.” In this exploratory analysis, significance was set at *p* ≤ 0.05. Because vascular burden may contribute to both brain health and frailty status, we performed additional sensitivity analyses by further adjusting for the prevalence of cardiovascular disease. Cardiovascular disease was defined based on self-reported medical history, medication use, or clinical exams (31). Values of FA and MD were in standardized Z scores due to small values. Because studies reported women tend to have higher prevalence of frailty than men, we tested sex differences by adding interaction terms of sex and frailty status in the model.

Due to the relatively older age of the frail group, we performed additional sensitivity analyses by selecting age-matched participants from the non-frail and pre-frail groups with a 1:2 ratio (non-frail: 2; pre-frail: 2; frail: 1). We repeated the analyses in this aged-matched sample to confirm the robustness of our findings obtained in the whole sample.

## Results

Participant characteristics are presented in Table 1. Based on the Fried frailty criteria, 54% of this study sample was categorized as “non-frail,” 41% was “pre-frail,” and 5% was “frail.” Those who were “frail” appeared to be older and more likely to be women, and have lower education, lower total brain volume, and higher white matter hyperintensity volume (Table 1).

**Table 1:** Participant characteristics (*n* = 670).

	**Non-frail (*n* = 362)**	**Pre-frail (*n* = 279)**	**Frail (*n* = 29)**	***p*-value**
	**mean** **±** **SD or** ***N*** **(%)**			
**Demographics**				
Age, years	70.2 ± 6.9	77.2 ± 7.8	81.3 ± 8.0	<0.001
Women	185 (51)	168 (60)	21 (72)	0.01
Black	90 (24)	66 (23)	7 (24)	0.94
APOE e4 carriers	92 (25)	63 (23)	6 (21)	0.64
Body mass index, kg/m^2^	27.2 ± 4.5	26.8 ± 4.4	27.2 ± 4.6	0.35
Years of education	17.7 ± 2.6	17.6 ± 2.9	17.0 ± 2.9	0.25
Cardiovascular disease	27 (7.5)	26 (9.3)	4 (13.8)	0.41
**Global neuroimaging markers**				
Total brain volume, cm^3^	1149 ± 111	1101 ± 110	1065 ± 125	<0.001
Total white matter hyperintensity volume, cm^3^, median (Q1-Q3)	3.3 (1.6-6.5)	5.4 (2.9-10.7)	8.7 (4.7-14.2)	<0.001
**Performance measures of frailty**				
Gait speed, m/sec	1.26 ± 0.17	1.06 ± 0.22	0.87 ± 0.14	<0.001
Muscle strength, kg	34.8 ± 9.4	26.0 ± 8.6	23.7 ± 8.1	<0.001
High intensity exercise, kcal/week, median (Q1-Q3)	1,655 (747-3045)	1,076 (111-2,297)	69 (29-314)	<0.001

*Values of total white matter hyperintensities in cm^3^ and high intensity exercise in kcal per week were presented as median (Q1-Q3) due to their skewed distribution. P-values were based on Spearman correlation coefficients for continuous variables and chi-square tests for categorical variables*.

After adjustment for age, sex, race, education, body mass index, and APOE e4 status, a higher frail score was associated with higher MD of the medial frontal cortex, putamen, caudate, thalamus, and anterior cingulate cortex (Table 2). A higher frail score was also associated with lower FA of the body of the corpus callosum, and superior fronto-occipital fasciculus (Table 3).

**Table 2:** Univariate and adjusted associations of frailty status (non-frail, pre-frail, frail) with mean diffusivity of gray matter regions of interest (ROIs).

	**Gray matter ROIs**	**Univariate associations**	**Adjusted associations**
		**β (95% CI)**	**β (95% CI)**
Frontal	Inferior frontal gyrus	**0.273 (0.144, 0.403)**	−0.116 (-0.244, 0.012)
	Superior frontal gyrus	**0.223 (0.094, 0.353)**	0.013 (-0.128, 0.153)
	Middle frontal gyrus	**0.332 (0.204, 0.460)**	−0.018 (-0.147, 0.112)
	Medial frontal cortex	**0.475 (0.349, 0.601)**	**0.146 (0.021, 0.270)**
	Supplementary motor area	**0.361 (0.234, 0.489)**	0.024 (-0.110, 0.158)
	Precentral gyrus	**0.254 (0.125, 0.384)**	−0.072 (-0.208, 0.064)
Parietal	Postcentral gyrus	**0.265 (0.135, 0.394)**	−0.049 (-0.184, 0.086)
	Precuneus	**0.451 (0.324, 0.577)**	0.015 (-0.105, 0.136)
	Angular gyrus	**0.359 (0.231, 0.487)**	0.002 (-0.118, 0.122)
	Supramarginal gyrus	**0.325 (0.196, 0.453)**	−0.048 (-0.169, 0.072)
Temporal	Entorhinal cortex	**0.319 (0.191, 0.448)**	0.041 (-0.091, 0.173)
	Parahippocampal gyrus	**0.465 (0.339, 0.591)**	0.074 (-0.055, 0.202)
	Inferior temporal gyrus	**0.417 (0.290, 0.544)**	0.016 (-0.098, 0.129)
	Superior temporal gyrus	**0.345 (0.217, 0.473)**	−0.056 (-0.172, 0.059)
	Middle temporal gyrus	**0.445 (0.319, 0.572)**	−0.006 (-0.109, 0.098)
Occipital	Inferior occipital gyrus	**0.353 (0.225, 0.481)**	−0.014 (-0.135, 0.107)
	Superior occipital gyrus	**0.283 (0.154, 0.413)**	−0.111 (-0.236, 0.014)
	Middle occipital gyrus	**0.319 (0.190, 0.447)**	0.024 (-0.102, 0.149)
Subcortical	Hippocampus	**0.536 (0.412, 0.661)**	0.079 (-0.030, 0.188)
	Putamen	**0.599 (0.476, 0.722)**	**0.209 (0.083, 0.335)**
	Caudate	**0.489 (0.363, 0.614)**	**0.157 (0.022, 0.292)**
	Thalamus	**0.498 (0.372, 0.623)**	**0.127 (-0.003, 0.258)**
Limbic	Anterior cingulate cortex	**0.512 (0.387, 0.637)**	**0.124 (0.004, 0.244)**
	Middle cingulate cortex	**0.304 (0.175, 0.433)**	−0.109 (-0.236, 0.018)
	Posterior cingulate cortex	**0.560 (0.436, 0.683)**	0.057 (-0.053, 0.167)

*Adjusted associations controlled for age, sex, race, education, BMI, and APOE e4. Bold numbers indicate associations at p ≤ 0.05. Mean diffusivity values were standardized Z scores*.

**Table 3:** Univariate and adjusted associations of frailty status (non-frail, pre-frail, frail) with fractional anisotropy of white matter regions of interest (ROIs).

**White matter ROIs**	**Univariate associations**	**Adjusted associations**
	**β (95% CI)**	**β (95% CI)**
Genu of the corpus callosum	**-0.511 (-0.643,−0.380)**	−0.098 (-0.232, 0.036)
Body of the corpus callosum	**-0.496 (-0.628,−0.363)**	**-0.164 (-0.305,−0.023)**
Splenium of the corpus callosum	**-0.421 (-0.554,−0.287)**	−0.114 (-0.258, 0.029)
Uncinate fasciculus	**-0.161 (-0.301,−0.022)**	−0.055 (-0.210, 0.100)
Superior longitudinal fasciculus	**-0.135 (-0.270, 0.0002)**	0.024 (-0.128, 0.176)
Inferior fronto-occipital fasciculus	**-0.287 (-0.422,−0.152)**	−0.071 (-0.222, 0.079)
Superior fronto-occipital fasciculus	**-0.355 (-0.488,−0.222)**	**-0.166 (-0.313,−0.018)**
Anterior limb of internal capsule	**-0.341 (-0.478,−0.205)**	−0.101 (-0.249, 0.047)
Posterior limb of internal capsule	**-0.084 (-0.221, 0.054)**	−0.021 (-0.171, 0.129)
External capsule	**-0.319 (-0.453,−0.185)**	−0.001 (-0.146, 0.143)
Posterior thalamic radiation	**-0.237 (-0.372,−0.101)**	−0.121 (-0.275, 0.033)
Anterior of corona radiate	**-0.496 (-0.627,−0.366)**	−0.091 (-0.224, 0.043)
Superior of corona radiate	**-0.219 (-0.355,−0.083)**	−0.064 (-0.217, 0.089)
Posterior of corona radiate	−0.032 (-0.168, 0.104)	0.005 (-0.148, 0.158)
Cingulum hippocampal part	**-0.211 (-0.346,−0.075)**	0.033 (-0.113, 0.179)
Cingulum cingulate part	**-0.270 (-0.404,−0.136)**	−0.008 (-0.150, 0.135)

*Adjusted associations controlled for age, sex, race, education, BMI, and APOE e4. Bold numbers indicate associations at p ≤ 0.05. Fractional anisotropy values were standardized Z scores*.

Compared to the non-frail group, the frail group had higher MD of the medial frontal cortex, putamen, caudate, thalamus, and anterior cingulate cortex; they also had lower FA of the body of the corpus callosum and superior fronto-occipital fasciculus at a marginal significance (*p* = 0.051) after full adjustment (Table 4). There were no differences in other ROIs between frail and non-frail groups (Table 4).

**Table 4:** Differences of neuroimaging markers identified in Tables 2, 3 between frailty status groups.

	**Regions of interest**	**Non-frail (*n* = 362)**	**Pre-frail (*n* = 279)**	**Frail (*n* = 29)**
		**Reference**	**β (95% CI)**	**β (95% CI)**
Gray matter mean diffusivity (higher=worse)	Medial frontal cortex	-	0.083 (−0.063, 0.228)	**0.482 (0.149, 0.815)**
	Putamen	-	**0.152 (0.005, 0.298)**	**0.595 (0.258, 0.932)**
	Caudate	-	0.086 (−0.071, 0.243)	**0.494 (0.134, 0.855)**
	Thalamus	-	0.020 (−0.132, 0.171)	**0.523 (0.176, 0.871)**
	Anterior cingulate cortex	-	0.012 (−0.127, 0.152)	**0.555 (0.236, 0.874)**
White matter fractional anisotropy (lower = worse)	Body of corpus callosum	-	−0.147 (−0.308, 0.015)	**−0.398 (−0.798, 0.002)**
	Superior fronto-occipital fasciculus	-	**−0.168 (−0.336**, **−0.001)**	**−0.400 (−0.803, 0.002)**

*Models were adjusted for age, sex, race, years of education, body mass index, scanner type, and APOE e4 status. Bold number reflects associations at p ≤ 0.05. Mean diffusivity and fractional anisotropy values were standardized Z scores*.

Compared to the non-frail group, the pre-frail group had significantly higher MD of the putamen, lower FA of the superior fronto-occipital fasciculus, and a trend toward lower FA of the body of the corpus callosum (*p* = 0.075) after full adjustment (Table 4). There were no differences of MD in other gray matter ROIs or FA in other white matter ROIs between pre-frail and non-frail groups (Table 4).

Additional adjustment for cardiovascular disease did not substantially alter these associations (Supplementary Table 2). Interaction terms of sex and frailty status were not statistically significant (data not shown).

In an age-matched sensitivity analysis adjusted for the same set of covariates except for age, results remained substantially similar except the association with FA of the superior fronto-occipital fasciculus. The magnitude of standardized regression coefficients was higher compared to the original analysis, although some of the comparisons showed a marginal significance given a smaller sample size. Specifically, the differences between non-frail and frail remained significant in MD of the medial frontal cortex, putamen, thalamus, and anterior cingulate cortex. Differences in MD of caudate and FA of the body of the corpus callosum showed a trend toward significance (*p* = 0.073 and 0.061, respectively) (Supplementary Table 3).

## Discussion

In this sample of cognitively normal older individuals, we demonstrate for the first time that both gray and white matter microstructure integrity is associated with frailty. Noteworthy, these neural correlates are localized in selected frontal and subcortical motor areas as well as the body of the corpus callosum. These results are not affected by adjustment for demographics and APOE e4 status. In particular, analyses conducted in an age-matched subsample confirmed the robustness of our findings.

Our DTI findings provide insight into mechanisms by which emerging brain microstructure damage, likely due to altered vascular integrity, may contribute to age-related frailty. Our findings may also explain frailty being a major risk factor of incident vascular dementia (32, 33). We advanced prior knowledge by including both gray and white matter microstructure, by determining the spatial distribution of microstructure with frailty, and by focusing on cognitively normal older adults.

Of white matter ROIs examined, we found that fractional anisotropy of the corpus callosum was associated with frailty, which was in line with one previous report (18). We further identified the body of corpus callosum being strongly associated with frailty status. We also observed a trend in superior fronto-occipital fasciculus, which is in line with previous findings (19). Notably, two previous DTI studies using tract-based spatial statistics report other DTI parameters, such as axial diffusivity (AD) and mean diffusivity (MD) of the anterior limb of internal capsule and superior corona radiata, differ by frailty status. Using the ROI approach, we also observed that AD and MD of these two ROIs were associated with the total frail score (data not shown).

We are the first to demonstrate microstructural characteristics of gray matter underlying frailty. Our findings concerning the spatial distribution of gray matter microstructure with frailty are in line with previous brain volumetric and β-amyloid findings (6, 12, 15, 34). Specifically, the medial frontal cortex is an identified area important for motor function and associated with lower extremity performance, perhaps due to its key role in executive function. We also observed a strong association of MD in the anterior cingulate cortex. The anterior cingulate includes the cingulate motor areas and is involved in locomotion. Its structure and function are associated with gait performance (35–37). The strong associations of MD in subcortical motor areas, including putamen, caudate, thalamus, highlight the disrupted subcortical integrity may precede frailty risk and the development of frailty. Notably, the thalamus has an important role in motor control and coordination and also has a strong structural and functional connection to the striatum of putamen and caudate.

We did not observe any association in the temporal lobe or specifically the hippocampus, which has been reported in previous brain volumetric studies by MRI and CT (6, 15, 34). This inconsistency may be due to differences in characteristics of study participants, imaging analytical approaches (voxel-based morphometry vs. ROIs), or frailty assessment.

Although differences in these microstructural neuroimaging markers were mostly present between frail and non-frail, there were also differences between prefrail and non-frail groups, such as putamen and the body of the corpus callosum. As the pre-frail stage is thought to be potentially revisable, strategies to slow or delay microstructural degradation in these selected areas in the absence of cognitive impairment might be considered.

Among limitations, we acknowledge the cross-sectional design which cannot establish the temporal sequence between brain microstructure and frailty. The BLSA sample tends to be healthier than the general population. The small number of frail cases may introduce potential bias. Using the Fried frailty criteria may have underestimated “pre-frail” and “frail” status in this sample. We would expect a stronger signal if multiple aspects of frailty are captured, especially in non-clinical, community-dwelling older populations. The modified definition items in the frailty criteria, such as leisure hour activities, may limit the ability to compare with other studies. This study has several strengths. First, the DTI atlas allowed us to investigate the detailed microstructural properties in both gray and white matter ROIs. Second, this sample community-dwelling older adults are well-characterized with rigorous adjudication of cognitive impairment, including dementia, and APOE e4 genotype. This allowed us to focus on frailty in the absence of cognitive impairment and to account for APOE e4 status. Third, the age-matched sensitivity analyses confirmed the robustness of our findings.

In conclusion, microstructural integrity of selected frontal and subcortical motor regions as well as the body of the corpus callosum may be critical neural substrates underlying frailty among cognitively normal older adults. Future studies on whether early changes in brain microstructure precedes the development of frailty are needed.

## Data Availability Statement

The raw data supporting the conclusions of this article will be made available by the authors, without undue reservation.

## Ethics Statement

The studies involving human participants were reviewed and approved by Institutional Review Board of the National Institute of Environmental Health Sciences. The patients/participants provided their written informed consent to participate in this study.

## Author Contributions

QT designed the study, participated in the quality control for the DTI data, performed statistical analyses, and drafted the manuscript. OW performed quality control for the DTI data, provided feedback and suggestions to the analyses, and critically evaluated the manuscript. BL developed DTI analytical approaches, provided feedback and suggestions to the analyses, and critically evaluated the manuscript. SR collected data, provided guidance on analyses, and critically evaluated the manuscript. LF designed the study, collected data, provided guidance on analyses, and critically evaluated the manuscript. All authors contributed to the article and approved the submitted version.

## Conflict of Interest

The authors declare that the research was conducted in the absence of any commercial or financial relationships that could be construed as a potential conflict of interest.
