# Supplementary material for: Microstructural Neuroimaging of Frailty in Cognitively Normal Older Adults
Source: Front Med (Lausanne). 2020 Oct 23;7:546344. doi: 10.3389/fmed.2020.546344 (PMC7645067; doi:10.3389/fmed.2020.546344)
Supplement: Supplementary file 1 [file Data_Sheet_1.docx]

**Supplementary Table 1. Frailty components and cutoffs.**

| **Components** | **Cutoff ^1^** | **BLSA cutoff** |
| --- | --- | --- |
| Weight loss | ≥ 5 kg (≥ 10 pounds) unintentional weight loss in the last year | Same |
| Weakness, grip strength | Men: ≤ 30 kg  Women: ≤ 20 kg | Same |
| Exhaustion | CES-D: “I felt everything I did was an effort”, or “I could not get going” with an answer of “3 to 4” or “5 to 7” days in the last week | Same |
| Slowness | Gait speed ≤ 0.8 m/sec | ≤ 1.0 m/sec |
| Low physical activity | Physical Activity Questionnaire for the Elderly (PAQE) ≤ 9.4 total score | High-intensity exercise ≤ 100 kcal/week |

**Supplementary Table 2. Cross-sectional associations of microstructural markers identified in Table 2 and 3 with frailty status**

|  | **Regions of interest** | **Non-frail**  **(n=362)** | **Pre-frail**  **(n=279)** | **Frail**  **(n=29)** |
| --- | --- | --- | --- | --- |
|  |  | **reference** | **β (95% CI)** | **β (95% CI)** |
| **Gray matter mean diffusivity, unitless**  **(higher=worse)** | **Medial frontal cortex** | **-** | 0.084 (-0.062, 0.229) | 0.480 (0.147, 0.813) |
|  | **Putamen** | **-** | 0.152 (0.005, 0.299) | 0.593 (0.256, 0.930) |
|  | **Caudate** | **-** | 0.086 (-0.071, 0.244) | 0.493 (0.132, 0.853) |
|  | **Thalamus** | **-** | 0.022 (-0.129, 0.173) | 0.518 (0.172, 0.863) |
|  | **Anterior cingulate cortex** | **-** | 0.013 (-0.126, 0.152) | 0.554 (0.235, 0.873) |
|  | | | | |
| **White matter fractional anisotropy, unitless**  **(lower=worse)** | **Body of corpus callosum** | **-** | -0.146 (-0.308, 0.016) | -0.396 (-0.797, 0.004) |
|  | **Superior fronto-occipital fasciculus** | **-** | -0.165 (-0.333, 0.002) | -0.391 (-0.794, 0.011) |

**with additional adjustment for cardiovascular disease.**

Note. Covariates included age, sex, race, education, body mass index, APOE e4 status, the scanner type, and cardiovascular disease.

**Supplementary Table 3. Age-matched sensitivity analyses for associations of frailty status with selected neuroimaging markers**

|  | **ROIs** | **Non-frail**  **(n=58)** | **Pre-frail**  **(n=58)** | **Frail**  **(n=29)** |
| --- | --- | --- | --- | --- |
|  |  | **reference** | **β (95% CI)** | **β (95% CI)** |
| **Gray matter mean diffusivity, unitless**  **(higher=worse)** | Medial frontal cortex | - | 0.085 (-0.271, 0.441) | 0.506 (0.024, 0.069) |
|  | Putamen | - | 0.360 (-0.057, 0.777) | 0.778 (0.266, 1.291) |
|  | caudate | - | 0.084 (-0.343, 0.511) | 0.480 (-0.045, 1.004) |
|  | Thalamus | - | 0.050 (-0.307, 0.407) | 0.728 (0.289, 1.167) |
|  | Anterior cingulate cortex | - | 0.044 (-0.291, 0.379) | 0.709 (0.297, 1.121) |
|  |  |  |  |  |
| **White matter fractional anisotropy, unitless**  **(lower=worse)** | Body of corpus callosum | - | -0.312 (-1.053, 0.105) | -0.513 (-1.053, 0.028) |
|  | Superior fronto-occipital fasciculus | - | -0.047 (-0.416, 0.323) | -0.246 (-0.717, 0.225) |

Note. Models were adjusted for sex, race, years of education, body mass index, the scanner type, and APOE e4 status. Mean diffusivity and fractional anisotropy values were standardized Z scores.

**References:**

1. Saum KU, Muller H, Stegmaier C, Hauer K, Raum E, Brenner H. Development and evaluation of a modification of the Fried frailty criteria using population-independent cutpoints. *J Am Geriatr Soc.* 2012;60(11):2110-2115.
